# Role of Inflammation in Vascular Disease-Related Perivascular Adipose Tissue Dysfunction

**DOI:** 10.3389/fendo.2021.710842

**Published:** 2021-08-11

**Authors:** Yaozhi Chen, Zeyu Qin, Yaqiong Wang, Xin Li, Yang Zheng, Yunxia Liu

**Affiliations:** ^1^Center for Cardiovascular Medicine, First Hospital of Jilin University, Changchun, China; ^2^Department of Respiratory Medicine, First Hospital of Jilin University, Changchun, China; ^3^Department of Endocrinology and Metabolism, First Hospital of Jilin University, Changchun, China

**Keywords:** perivascular adipose tissue, vascular diseases, inflammation, endocrine, crosstalk

## Abstract

Perivascular adipose tissue (PVAT) is the connective tissue around most blood vessels throughout the body. It provides mechanical support and maintains vascular homeostasis in a paracrine/endocrine manner. Under physiological conditions, PVAT has anti-inflammatory effects, improves free fatty acid metabolism, and regulates vasodilation. In pathological conditions, PVAT is dysfunctional, secretes many anti-vasodilator factors, and participates in vascular inflammation through various cells and mediators; thus, it causes dysfunction involving vascular smooth muscle cells and endothelial cells. Inflammation is an important pathophysiological event in many vascular diseases, such as vascular aging, atherosclerosis, and hypertension. Therefore, the pro-inflammatory crosstalk between PVAT and blood vessels may comprise a novel therapeutic target for the prevention and treatment of vascular diseases. In this review, we summarize findings concerning PVAT function and inflammation in different pathophysiological backgrounds, focusing on the secretory functions of PVAT and the crosstalk between PVAT and vascular inflammation in terms of vascular aging, atherosclerosis, hypertension, diabetes mellitus, and other diseases. We also discuss anti-inflammatory treatment for potential vascular diseases involving PVAT.

## Introduction

The vascular system is a highly branched network lined with endothelial cells (ECs) and vascular smooth muscle cells (VSMCs), which can provide oxygen and nutrition for tissues. The regulation of vascular function in response to changing metabolic needs is essential for the maintenance of normal tissue and organ functions; it is also important for health preservation and disease prevention (1). Vascular diseases caused by vascular injury and dysfunction are among the top five causes of death among non-communicable diseases worldwide; these diseases influence various other diseases, such as heart diseases, nervous system diseases, and metabolic disorders (2). Vascular homeostasis is regulated by many factors, among which perivascular adipose tissue (PVAT) plays an important role in the pathogenesis of vascular diseases.

PVAT, which surrounds most blood vessels (except cerebral vessels) (3), is a connective tissue composed of adipocytes, preadipocytes, mesenchymal stem cells, fibroblasts, inflammatory cells (macrophages, lymphocytes, and eosinophils), vascular cells, and nerves; these cells form adipose tissue microvasculature (4). PVAT is characterized by a reduced degree of differentiation, compared with classical visceral fat (PVAT more closely resembles preadipocytes); moreover, it exhibits a tendency to release pro-inflammatory factors and growth factors (5). Because of the anatomical characteristics of its adjacent vessel walls, PVAT provides mechanical support in the vascular system, particularly during adjacent tissue contraction. Furthermore, PVAT releases various factors, including adipokines and cytokines/chemokines. Through paracrine/endocrine mechanisms, these factors can directly diffuse or reach the vascular endothelial layer through blood vessels or a network of small mediators that connects the middle layer and lower adventitia. Additionally, these factors regulate vascular tension, cell proliferation, and cell migration; exhibit considerable influence on vascular homeostasis and function; and demonstrate both protective and harmful effects on the vascular system, according to pathophysiological characteristics present in the tissue microenvironment.

Under physiological conditions, PVAT has anti-inflammatory effects, improves free fatty acid metabolism, and regulates vasodilation. However, in the event of vascular pathology, PVAT increases in volume and becomes dysfunctional. This leads to changes in cell composition and molecular characteristics, as well as extensive secretion of pro-inflammatory and anti-vasodilation factors; it also promotes the infiltration of inflammatory immune cells and local oxidative stress, triggering vascular wall “from the outside to the inside” pathological signal, thereby causing VSMC and EC dysfunction (6, 7). The specific mechanisms and characteristics of PVAT dysfunction may differ among vascular diseases, despite important similarities with respect to inflammation characteristics. Inflammation is also an important pathophysiological event in vascular aging, atherosclerosis, hypertension, diabetes mellitus (DM), and other vascular diseases (8, 9). Therefore, the pro-inflammatory crosstalk between PVAT and blood vessels may comprise a novel therapeutic target for the prevention and treatment of vascular diseases. In this review, we summarize the latest findings regarding PVAT function and inflammation in various pathophysiological contexts and discuss anti-inflammatory treatments for potential PVAT-related vascular diseases.

## Secretory Functions of PVAT

**Figure 1:**
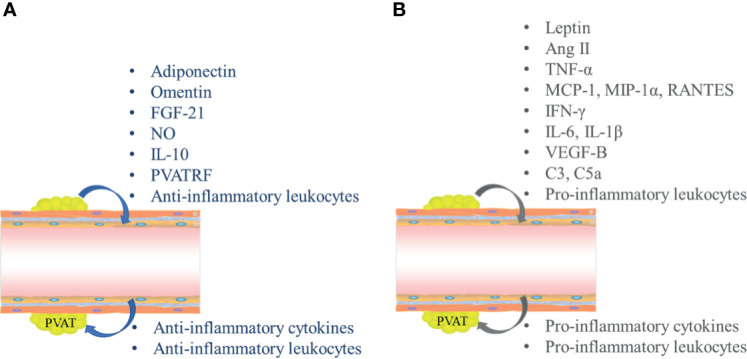
Secretory functions mediate inflammatory crosstalk between perivascular adipose tissue (PVAT) and blood vessels. **(A)** Interactions between PVAT and blood vessels in normal physiological conditions. **(B)** Crosstalk between PVAT and blood vessels in pathological conditions. FGF-21, fibroblast growth factor-21; NO, nitric oxide; IL, interleukin; PVATRF, PVAT-derived relaxing factor; TGF-β1, transforming growth factor-β1; Ang II, angiotensin II; TNF-α, tumor necrosis factor-α; MCP-1, monocyte chemoattractant protein-1; MIP-1α, macrophage inflammatory protein 1α; IFN-γ, interferon γ; VEGF-B, vascular endothelial growth factor B.

PVAT is considered an important endocrine tissue for the maintenance of intravascular stability. Although most inflammation in PVAT is attributed to infiltration by macrophages and T cells, PVAT-related regulation of vascular function depends largely on its secretory functions (**Figure 1**). Dysfunctional adipocytes themselves exhibit pro-inflammatory phenotypes and may play important roles in triggering and spreading inflammation within PVAT (10). Similar to other adipose tissues, PVAT secretes many adipose tissue-specific adipokines, chemokines, and growth factors; these directly affect the functions of adjacent blood vessels in a paracrine manner and can also reach the lumens of adjacent blood vessels, then have various downstream effects. PVAT affects tension and endothelial functions throughout the vascular bed in a vascular secretion manner, thus triggering and coordinating the infiltration of inflammatory cells (e.g., T cells, macrophages, dendritic cells, B cells, and NK cells) (11).

Under physiological conditions, PVAT mainly secretes anti-inflammatory adipokines, such as adiponectin (APN), omentin, fibroblast growth factor-21 (FGF-21), and nitric oxide (NO) (**Figure 1A**). In the context of vascular dysfunction, PVAT mostly produces and releases pro-inflammatory adipokines, such as leptin, tumor necrosis factor-α (TNF-α), monocyte chemoattractant protein-1 (MCP-1, also known as CCL2), RANTES (Chemokine C-C motif ligand 5, CCL5), interleukin-6 (IL-6), and interleukin-1β (IL-1β); all of these factors can directly affect VSMCs and ECs, thus triggering and coordinating vascular inflammation (**Figure 1B**) (5, 9).

### PVAT and Anti-Inflammatory Cytokines

Under physiological conditions, PVAT releases various anti-inflammatory factors, including APN. The main biological functions of APN include promotion of fatty acid biosynthesis and inhibition of gluconeogenesis in liver, enhancement of glucose uptake in skeletal muscle, improvement of systemic insulin resistance, and prevention of systemic atherosclerosis by increasing fatty acid oxidation (12).

The effects of APN-mediated anti-inflammatory action on vascular function have been elucidated in multiple vascular diseases. Compared with mice that were fed a regular chow diet, the anti-contractile effect of PVAT was significantly reduced in non-endothelial aortic rings in mice that were fed a high-fat diet, partly because of reduced APN release from PVAT, associated with AMPK dysfunction and/or PVAT inflammation (13–16). APN expression is reportedly downregulated in desoxycorticosterone acetate (DOCA)‐salt hypertensive mice because of complement activation in PVAT; this effect can be reversed by macrophage depletion (17). Transplanted PVAT exhibits reduced expression of APN, thereby aggravating endothelial dysfunction through an inflammatory response (18). Exogenous APN can restore aortic anti-contractile activity of adult male offspring in mice exposed to gestational intermittent hypoxia (19). Upregulation of the heme oxygenase-1/APN axis in PVAT mediates anti-contractility-related Irisin improvement in the thoracic aorta of obese mice (15, 20). Chronic APN therapy can inhibit chemokine and pro-inflammatory adipokine expression patterns in PVAT in both aged rats and rats that were fed a high-fat diet, thus improving endothelial dysfunction in these models (21). PVAT around the coronary artery, collectively classified as epicardial adipose tissue (EAT), is closely related to the occurrence and development of coronary atherosclerotic lesions, as well as plaque stability. Angiotensin converting enzyme 2 (ACE2)-knockout mice that were fed a high-fat diet exhibited increased EAT inflammation; this was associated with decreased cardiac APN, decreased AMPK phosphorylation, increased cardiac steatosis and lipotoxicity, and myocardial insulin resistance, which exacerbated cardiac functional damage (22). In EAT from patients with coronary heart disease (CHD), the levels of APN decreased, while the levels of IL-6, TNF-α, and Toll-like receptor 4 increased. APN administration has been shown to prevent atherosclerosis by reducing the production of TNF-α in macrophages and reactive oxygen species (ROS) in endothelial cells; it also increases endothelial cell migration and angiogenesis (23). Samples from humans indicate that type 2 diabetes (T2DM) is closely related to hypoadiponectin, suggesting that the APN signaling pathway can serve as a new route for vascular protection in blood vessels and PVAT. However, the molecular mechanism by which APN reduces vascular inflammation remains unclear. Inhibition of nuclear factor kappa-B (NF-κB) signaling (which regulates several pro-inflammatory genes) and its transcription factors may be another mechanism by which APN alleviates vascular dysfunction (24).

Omentin (also known as intelectin-1, lactoferrin receptor, or endothelial lectin) has a positive effect on vascular inflammation. By inhibiting the thioredoxin-interacting protein/nucleotide-binding oligomerization domain-like receptor family pyrin domain-containing 3 signaling pathway, omentin can reduce the production of pro-inflammatory cytokines (e.g., TNF-α, IL-6, and IL-1β) and increase the production of anti-inflammatory cytokines (e.g., APN and IL-10) in obese mouse adipose tissue, as well as macrophages co-cultured with lipopolysaccharides (25, 26). The anti-contractile effect of PVAT in a physiological environment was lost in patients with T2DM, although it partially recovered after treatment with omentin-1, the main cyclic forms of omentin (27). Furthermore, omentin-1 treatment significantly improved the pro-inflammatory and pro-oxidant PVAT phenotype (i.e., through reduction of C-reactive protein and nitrotyrosine levels), suggesting that omentin-1 could improve endothelial dysfunction in T2DM patients by improving dysfunction PVAT; it also has the potential to treat T2DM-related vascular complications (28). In patients with atrial fibrillation and valvular heart diseases, the expression of omentin was downregulated in EAT and right atrial appendage tissue (29). Importantly, the expression of omentin in EAT was lower in patients with CHD than in patients without CHD; the expression of omentin in EAT was lower around stenotic segments of coronary artery than around non-stenotic segments (30). However, another study showed that, compared with the control group, the expression of omentin in EAT increased in patients with CHD, despite reduction of its circulating level; this finding suggested that omentin may play a local role in the development of CHD (31).

FGF-21, a member of the fibroblast growth factor family, is an important endocrine regulator that mainly acts through induction of weight loss and management of insulin signaling, as well as management of glucose and lipid metabolism (32). It also has important anti-inflammatory roles in various tissues/cells, such as obese adipose tissue, cardiac tissue, and macrophages (33–37). FGF-21 gene expression was reportedly reduced in EAT from patients with multivessel CHD associated with T2DM (38), while FGF-21 expression was enhanced in EAT from patients undergoing cardiac surgery, suggesting that FGF-21 has a protective effect against cardiac surgery-related inflammation (39). Therefore, anti-inflammatory pathways related to PVAT may comprise novel targets for the prevention and treatment of various vascular diseases.

### PVAT and Pro-Inflammatory Cytokines

Leptin is another rich adipokine released by adipose tissue (including PVAT), which was the first adipokine reported in the literature. Under physiological conditions, leptin mainly relies on hypothalamus and sympathetic nerve signaling to reduce appetite, increase energy consumption, and regulate glucose homeostasis, independent of insulin (40). Leptin resistance is associated with the development of hypertension and insulin resistance (40), and inflammation is an important contributor to leptin resistance (41). Classically, leptin is regarded as a pro-inflammatory cytokine, which has structural homology with other cytokines such as TNF-α and IL-6 (41, 42). Leptin plays a direct role in inflammation by inducing monocytes, leukocytes, and macrophages to produce IL-6, TNF-α, and IL-12; thus, it increases the production and migration of ROS in monocytes, as well as the production of chemokine ligands by macrophages (42, 43).

In obese sedentary mice, PVAT ring-deficient mice exhibited lower levels of circulating glucose, insulin, resistin, leptin, and TNF-α; they also demonstrated abnormal endothelial function in thoracic aorta (44). The reduction of leptin expression in PVAT may inhibit neointimal hyperplasia and vascular remodeling by inhibiting monocyte migration and VSMC proliferation (45). Gene expression profiling showed that the expression levels of IL-1β, IL-6, and leptin in patients with CHD were significantly higher in PVAT around the coronary artery than in PVAT inside the thoracic artery (46). In addition, data from patients undergoing coronary artery bypass grafting indicated that the leptin–inflammation–fibrosis–hypoxia axis plays a key role in coronary atherosclerosis pathogenesis. Compared with PVAT surrounding the anti-atherosclerotic internal mammary artery, leptin expression was significantly increased in “cardiac” PVAT surrounding the aortic root and coronary arteries (C-PVAT). This increased expression was accompanied by more obvious angiogenesis and inflammation, indicating significant increases in the numbers of platelets, endothelial cell adhesion molecule 1-positive vessels, and CD68-positive macrophages, as well as greater degrees of fibrosis and hypoxia, which may lead to an enhanced coronary atherosclerotic plaque load (47). Increased expression levels of hypoxia inducible factor-1α and Fos-like antigen 2 were observed in C-PVAT; these factors reportedly enhance leptin gene transcription (47). The findings thus far suggest that PVAT plays an important role in promoting vascular inflammation through leptin secretion.

Notably, adipocytes and macrophages in PVAT also secrete large amounts of TNF-α; these levels are higher in obese animals and people than in lean animals and lean people. TNF-α induces aortic intima-media thickening through PVAT inflammation (48). TNF-α has pro-inflammatory effects and can promote the production of other pro-inflammatory factors (e.g., IL-6, leptin, and resistin). Furthermore, MCP-1 produced by adipocytes has been identified as a potential factor for macrophage infiltration into adipose tissue (49, 50). The increased expression levels of MCP-1 and TNF-α in transplanted PVAT tissue can aggravate endothelial dysfunction and atherosclerosis in distant vessels by enhancing the inflammatory response (18). In addition, RANTES (produced by T cells, macrophages, VSMCs, ECs,and PVAT adipocytes (51–53)) is a key factor for leukocyte recruitment to sites of inflammation or infection (54). Increased RANTES expression levels in hypertensive PVAT induce T-cell chemotaxis and vascular accumulation of T cells that express RANTES receptors (55). In addition, PVAT secretes free fatty acids, resistin, visfatin, and other pro-inflammatory adipokines, which participate in the occurrence and development of vascular diseases (56–58).

## Roles of PVAT in Vascular Disease

Morphological, structural, and functional changes of PVAT have been investigated in major vascular lesions associated with vascular diseases such as vascular aging, atherosclerosis, hypertension, and DM-related vascular dysfunction (**Table 1**).

**Table 1:** Central roles of PVAT inflammation in vascular diseases.

	Vessels	PVAT	Reference
**Vascular aging**	Increased tunica media thickness Increased oxidative stress and inflammation (ET-1, iNOS, COX2) VSMCs proliferation Increased perivascular fibrosis Increased arterial stiffness	PVAT was hypertrophic and the average area of single adipocyte was significantly increased The differentiation ability of PVASCs decreased Increased proinflammatory mediators (TNF-α, IL-6, eotaxin, MIP-1α, MCP-1 and RANTES) Reduction of ant-inflammatory mediators (APN) The function of anti- vasoconstriction is weakened The infiltration of macrophages and natural killer cells	(59–68)
**Atherosclerosis**	The infiltration of macrophages, T cells and dendritic cells increased Plaque volume increased, internal lipid increased, high macrophage density and fibrin deposition Increased proinflammatory mediators Increased perivascular inflammation	The size of adipocytes was smaller and the differentiation phenotype was less Increased proinflammatory mediators (TNF-α, IL-6, IL-1β, MCP-1, resistin, and osteoprotegerin) Reduction of ant-inflammatory mediators (APN) The infiltration of macrophages, T cells and dendritic cells increased B-1 cells and their secretion of anti-atherosclerotic IgM decrease	(10, 18, 69–77) (67, 78–86)
**Hypertension**	Increased proinflammatory mediators (MCP-1, RANTES and MIP-1α) Endothelial dysfunction Increased vascular tone Systolic and diastolic blood pressure increased vascular hypertrophy and fibrosis Increased perivascular inflammation	The function of anti- vasoconstriction is weakened Decreased production of vasodilator factor Increased angiotensin II secretion Increased proinflammatory mediators (IFN-γ, RANTES, C3, C5a, MCP-1, TNF-α, IL-6, MIP-1α) Reduction of ant-inflammatory mediators (APN) The infiltration of macrophages, T cells and dendritic cells increased The number of eosinophils decreased	(17, 55, 68, 72, 87–97)
**Diabetes mellitus related vascular dysfunction**	Increased insulin resistance Impaired vasodilation and vascular remodeling mediated by insulin The adhesion ability of endothelial cells to lymphocytes increased	PVAT phenotype changed to pro-inflammatory, pro oxidative and pro vasoconstrictive state Increased infiltration of M1 macrophages and dendritic cells Overproduction of proinflammatory cytokines(IFN-γ, TNF-α, and IL-6) Reduction of anti-inflammatory cytokines (IL-10 and APN)	(98–102)
**Abdominal aortic aneurysm**	Recruitment of inflammatory cells (macrophages, lymphocytes, and mast cells) in vascular wall Increased expression of perivascular inflammatory factors Enhanced leukocyte- fibroblast interaction in adventitia Migration and proliferation of adventitial fibroblasts increased	Increased PVAT deposition Co-localization of PVAT inflammation and abdominal aortic aneurysm Increased gene expression of proinflammatory factors (IL-8, PTPRC, LCK, and CCL5) Decreased expression of anti-inflammatory PPARγ	(103–111)

The table lists changes in PVAT and vascular inflammation during the onset of vascular aging, atherosclerosis, hypertension, diabetes mellitus-related vascular dysfunction and abdominal aortic aneurysm (note that the anti-atherosclerotic effects of healthy PVAT are not listed). ET-1, endothelin-1; iNOS, inducible nitric oxide synthase; COX2, cyclooxygenase 2; VSMCs, vascular smooth muscle cells; PVASCs, resident stromal cells in PVAT; APN, adiponectin; PTPRC, protein tyrosine phosphatase receptor type C; LCK, lymphocyte-specific protein tyrosine kinase; PPARγ, peroxisome proliferator-activated receptor gamma.

### Vascular Aging

Aging is an independent risk factor for vascular diseases. Vascular aging is mainly characterized by blood vessel-related structural changes and dysfunction that increase with age, which culminate in age-related vascular diseases (112–114). In such diseases, the vascular wall exhibits a pro-inflammatory microenvironment associated with low-grade perivascular inflammation, which is characterized by increased secretion of pro-inflammatory cytokines and chemokines, as well as enhanced infiltration of immune cells (4). These changes promote vascular dysfunction, hinder cell metabolism, increase cell apoptosis, and contribute to the onset of vascular diseases (115). In this context, PVAT is a key factor that affects vascular and perivascular inflammation during aging (**Table 1**).

There is increasing evidence that age can affect PVAT morphology and function, increase PVAT-related inflammation, and affect the corresponding vascular activity. In rats, aging reportedly attenuated the anti-contractile effect of PVAT around the thoracic aorta, while reducing the amount of brown adipose tissue-like PVAT (59). In the mesenteric arteries of SHRSP.Z-Leprfa/IzmDmcr rats (SHRSP.ZF) with metabolic syndrome, vascular dysfunction is compensated by a PVAT-dependent mechanism, which disappears with age (60). Compared with young C57BL/6JRj mice, middle-aged mice showed more PVAT hypertrophy (61); furthermore, the mean single adipocyte area in PVAT was significantly increased, while the expression level of protein inhibitor of activated signal transducer and activators of transcription 1 (a key negative regulator of inflammation) was decreased. These effects may contribute to age-related vascular diseases (61).

With increasing age, resident stromal cells in PVAT (PVASCs) exhibit decreasing differentiation ability, which contributes to neointimal hyperplasia and vascular remodeling after PVAT transplantation into carotid artery. This may be caused by the loss of PGC1α in aged PVASCs, which can be improved by overexpression of PGC1α (62). Senescence-accelerated prone mice (SAMP8), a mouse model of aging, shows vascular dysfunction associated with hypertension and cognitive decline (116). Compared with control senescence-accelerated resistant mice (SAMR1), aged SAMP8 mice reportedly demonstrated the lack of an anti-vasoconstrictive effect of PVAT; they also exhibited increased tunica media thickness, decreased APN expression, and enhanced expression levels of vascular markers of inflammation (e.g., endothelin-1, inducible nitric oxide synthase, and cyclooxygenase 2) (63).

Arterial stiffness is an inevitable result of aging. Local PVAT homeostasis, especially inflammation in PVAT, is associated with the development of age-related arterial stiffness. Loss of functional PVAT can enhance arterial stiffness in aging mice; furthermore, aged C57BL/6J mice that were fed a high-fat diet demonstrated significant induction of PVAT hypertrophy and enhancement of arterial stiffness. This change is related to the low level of mitoNEET expression in PVAT, which increases the expression of pro-inflammatory genes (64). In addition, older arteries are more susceptible to obesity-induced aging, compared with younger arteries (65). Aging aggravates obesity-induced PVAT inflammation, promotes secretion of pro-inflammatory factors by PVAT (including cytokines such as TNFα and IL-6, as well as chemokines such as eotaxin and MIP-1α), and reduces APN secretion, thus increasing vascular oxidative stress and inflammation in a paracrine manner, and stimulating VSMC proliferation (65, 66).

The effect of PVAT on vascular senescence depends on its secretion, as well as the presence of inflammatory cells. Studies in humans have shown that age and body mass index are associated with the density of CD68-positive macrophages in PVAT (67). In spontaneously hypertensive rats, aging is associated with increased numbers of infiltrating leukocytes, macrophages, and natural killer cells in PVAT, accompanied by gradual elevation of blood pressure. Dual pharmacological inhibition of Nox1 and NOX4 increases blood pressure and leads to the accumulation of immune cells in PVAT. These effects are related to increased expression levels of MCP-1 and RANTES in PVAT, which lead to enhancement of perivascular fibrosis and acceleration of vascular aging (68). Overall, the existing evidence shows that PVAT mediates changes associated with vascular aging by enhancing infiltration of multiple inflammatory cells and release of various pro-inflammatory factors.

### Atherosclerosis

Vascular diseases (e.g., myocardial infarction and cerebral infarction) are often caused by atherosclerosis, a vascular disease with robust inflammation, which is characterized by the accumulation of lipids, diseased cells, and necrotic debris. Proinflammatory leukocytes and cytokines play important roles in various stages of atherosclerotic plaque formation (117). There is increasing evidence that perivascular inflammation contributes to multiple stages of atherosclerosis; notably, PVAT plays an important role in triggering adventitial inflammation in atherosclerosis. Furthermore, PVAT promotes atherosclerosis in basic vessels through “from the outside to the inside” signal transduction. However, PVAT exhibits a nonuniform role in the development of atherosclerosis. PVAT may have dual effects (i.e., both pro- and anti-atherosclerosis), which may influence balance in the local environment.

Healthy PVAT plays a protective role in regulating metabolism, inflammation, and function in nearby blood vessels. Healthy PVAT may contain immune cells that impede the development of atherosclerosis (118). The absence of PVAT can lead to enhanced macrophage infiltration and increased pro-inflammatory cytokine production in the aortic perivascular area, thus exacerbating vascular inflammation and atherosclerotic lesions in aortic wall tissue (119). In addition, PVAT is the main source of aorta-associated B lymphocytes. Many of these B cells belong to the anti-atherosclerotic B-1 subgroup; IgM secreted by this subgroup of B cells can reduce the effects of pro-inflammatory cytokines produced by M1 macrophages. Notably, the ratio of B-1/B-2 cells is reportedly 10-fold higher in PVAT than in spleen or bone marrow, indicating an important anti-inflammatory effect of PVAT (120).

The number of B-1 cells secreting anti-atherosclerotic IgM is reportedly reduced in PVAT from apolipoprotein E-/- (ApoE-/-) mice, which significantly aggravates atherosclerosis in the aorta and coronary artery (69). A systemic endocrine mechanism also mediates the anti-atherosclerotic effect of PVAT. The transplantation of PVAT from thoracic aorta of wild mice was able to reduce the atherosclerotic plaque size in suprarenal aorta of ApoE-/- mice that were fed a high-cholesterol diet. This anti-atherosclerotic effect was mediated by a transforming growth factor-β1-induced anti-inflammatory response, which may involve alternatively activated macrophages (121). In addition, APN derived from PVAT can inhibit carotid collar-induced carotid atherosclerosis by promoting macrophage autophagy (122).

In the context of chronic hyperthermia, PVAT dysfunction exacerbates atherosclerosis and increases the risk of plaque rupture. In dysfunctional PVAT, the secretion of anti-inflammatory factors (e.g., APN) is decreased, while the secretion of pro-inflammatory cytokines is increased; thus, the distribution of pro-inflammatory and anti-inflammatory immune cells is unbalanced. These changes lead to the enhancement of local inflammation, aggravating the development of atherosclerosis (**Table 1**).

Pathological conditions (such as altered expression of angiotensin II [Ang II] or pro-atherosclerotic factors) increase the dedifferentiation of PVAT adipocytes (70, 71). In larger vessels associated with atherosclerosis, adipocytes in PVAT are usually smaller and exhibit a less differentiated phenotype (10, 72, 73). Inflammation in PVAT and adventitia occurs prior to endothelial dysfunction and atherosclerotic plaque formation (74). In human aorta, PVAT accumulates in sites where atherosclerosis can easily form, while inflammatory cells concentrate in PVAT at the edge of adventitia and secrete chemokines that can attract monocytes and T cells to the adventitial interface, suggesting that PVAT promotes vascular inflammation (75). Compared with non-diseased aorta, inflammatory cells exhibited significantly increased infiltration in PVAT around atherosclerotic aorta (123). During the onset of atherosclerosis in ApoE-/- mice, macrophages, T cells, and dendritic cells were recruited into the adventitia and PVAT (75, 76), in a manner influenced by age (75). In addition, compared with subcutaneous adipose tissue transplantation in mice, carotid artery transplantation of PVAT reportedly causes large lipid rich atherosclerotic lesions in thoracic aorta, as well as high macrophage density and fibrin deposition. The inhibition of leukocyte ligand P-selectin glycoprotein ligand 1 may provide a therapeutic method to reduce the effects of PVAT inflammation on atherosclerosis (77). PVAT expansion and inflammation in obesity can remotely induce endothelial dysfunction and aggravate atherosclerosis. Transplantation of PVAT from the abdominal aorta of mice that were fed a high-fat diet promoted inflammation (increased expression of TNF-α and MCP-1; decreased expression of APN), endothelial dysfunction, and atherosclerosis in thoracic aorta, suggesting that enhanced inflammation is the potential mechanism by which PVAT exhibits a distal vascular effect (18).

Data from human samples showed that the densities of B lymphocytes and macrophages in PVAT around atherosclerotic plaque increased with plaque size; the corresponding inflammation increased with increasing coronary artery occlusion (67). The number of macrophages in PVAT was also associated with the number of immune cells in plaque (78–80). In addition, the density of macrophages was higher in PVAT near unstable plaque than in PVAT near the stable plaque. The inflammation was stronger in PVAT near stenotic sites and acute lesions than in adipose tissue distant from lesions, in the absence of atherosclerosis (67).

Pro-atherosclerotic mediators derived from dysfunctional PVAT may comprise another mechanism underlying human vascular atherosclerosis (81). Analysis of dysfunctional PVAT has revealed upregulated expression of pro-inflammatory genes, as well as downregulated expression of anti-inflammatory adiponectin (82–84). EAT from patients undergoing coronary artery bypass grafting showed significantly higher levels of chemokines (MCP-1) and pro-inflammatory cytokines (IL-1β, IL-6, and TNF-α), compared with levels in subcutaneous adipose tissue from the same patients (84). Furthermore, the expression of anti-inflammatory APN was significantly lower in EAT samples from patients with severe coronary atherosclerosis than in EAT samples from patients without coronary atherosclerosis (83), suggesting an inflammation imbalance in PVAT from patients with atherosclerosis. The colocalization of macrophages and resistin (an adipokine that can enhance endothelial cell permeability *in vitro*) in human PVAT indicates that PVAT may participate in the pathogenesis of atherosclerosis through various mechanisms (82, 85). Osteoprotegerin, a member of the TNF-related family, is associated with atherosclerotic progression and increased instability; its expression is strongly upregulated in human perivascular adipocytes (124).

Importantly, vascular wall-associated inflammation also affects the dynamic balance of PVAT. In the presence of coronary artery inflammation and atherosclerosis, the release of pro-inflammatory mediators from the vascular wall to the surrounding PVAT leads to altered adipocyte differentiation and intracellular lipid formation, which greatly influences cardiovascular diagnosis and contributes to distinctive imaging findings (86). Noninvasive detection of PVAT can provide structural information and distinguish unstable atherosclerotic lesions. In postmortem studies of human patients, atherosclerotic plaque size and complex lipid core composition were positively associated with PVAT volume and macrophage infiltration (79). Because PVAT inflammation is related to disordered adipocyte differentiation and reduced lipid content in adipocytes, Antonopoulos et al. examined the perivascular CT fat attenuation index (FAI), a water to fat ratio index with good sensitivity and specificity in the differential diagnosis of PVAT inflammation (86). Importantly, they found that the perivascular FAI was greater in unstable plaque than in stable plaque; it was greatest near inflamed coronary arteries. PVAT imaging can provide spatial location information regarding the human coronary arteritis microenvironment, which enables early identification of high-risk plaques and may facilitate further treatment.

### Hypertension

Inflammation is an important factor involved in hypertension, which involves high blood pressure and can cause both end organ damage and dysfunction (125, 126). The main site of initial inflammation in hypertension is reportedly within PVAT and the PVAT/adventitia boundary (55, 127). Inflammation leads to the loss of the anti-contractile effect of PVAT, potentially because of adipose tissue dysfunction (128). The production of vasodilators derived from PVAT adipocytes decreases during inflammation, while pro-inflammatory adipokines increasingly infiltrate into the adjacent vascular system (5); these changes enhance vascular inflammation and vascular resistance (68). PVAT inflammation leads to vascular dysfunction in the context of hypertension. Various inflammatory cells participate in this process, which is mediated by a series of cytokines and chemokines; for example, interferon-γ is produced by CD8+ cells infiltrating PVAT (55, 87), RANTES mediates the infiltration of T cells into perivascular space (55), and complement C5a mediates decreased APN production (17). These inflammation-related changes exacerbate the pro-inflammatory crosstalk and dysfunction between PVAT and hypertensive vessels (**Table 1**).

The infiltration and activation of macrophages dispersed in PVAT are important contributing factors in hypertension-related inflammation. The expression of complement C3 is reportedly increased in PVAT from DOCA‐salt hypertensive mice (88), resulting in increased expression of pro-inflammatory M1 macrophage phenotype markers and decreased expression of anti-inflammatory M2 macrophage phenotype markers. Bone marrow-specific C3 deficiency significantly improved DOCA-salt–induced hypertensive vascular hypertrophy and fibrosis (89). Further studies in DOCA‐salt hypertensive mice showed that the recruitment of macrophages in PVAT promotes complement activation, induces perivascular inflammation, and increases the production of TNF-α, thereby causing APN downregulation. This is a potential risk factor for hypertension-related vascular inflammation and injury (17), which may be particularly important when considering treatment methods for hypertension-related vascular injury. Moreover, in mice with spontaneous hypertension induced by perilipin-1 deletion, PVAT exhibited reduced APN expression, whereas it exhibited enhanced expression of MCP-1, TNF-α, and IL-6; additionally, the anti-contractile effect of PVAT was lost. These effects were associated with an increased pro-inflammatory response, as well as higher systolic and diastolic blood pressures in aorta (90).

Increased activation of the renin–angiotensin–aldosterone system (RAS) is an important factor in hypertension pathogenesis. With the exception of renin, almost all components of the RAS system are expressed in PVAT, implying key roles in the regulation of hypertension-related perivascular inflammation (72, 129). Lee et al. found that the release of PVAT-derived relaxing factor (PVATRF) from PVAT in spontaneously hypertensive rats was significantly reduced, while the release of Ang II was enhanced (91). In Ang II-induced hypertensive mice, the numbers of leukocytes, T cells, macrophages, and dendritic cells in PVAT were significantly increased (55, 92). In those mice, Ang II significantly increased the expression levels of MCP-1, RANTES, and macrophage inflammatory protein 1α (MIP-1α, also known as CCL3) in aorta and PVAT. Furthermore, the activation of angiotensin type 1 receptor (AT1 receptor) in PVAT promotes vascular inflammation and endothelial dysfunction (93, 94). Aldosterone may directly promote a pro-inflammatory phenotype in PVAT. Macrophage infiltration and increased C5a expression were detected in adipose tissue from patients with aldosterone-secreting adenoma; these findings were associated with decreased APN expression (17).

Hypertension was more common in obese individuals than in lean individuals. The progress of hypertension is related to the immune response of adipose tissue (130). The anti-vasoconstrictive properties of healthy PVAT are eliminated in obesity, resulting in increased arterial tension, which is a key mechanism of obesity-related hypertension and vascular dysfunction. Macrophage infiltration in PVAT plays a key role in obesity-related hypertension (95). In mice that were fed a high-fat diet, macrophages accumulated in fat PVAT around the thoracic aorta or mesenteric artery. The absence of class A1 scavenger receptor, a key pattern recognition receptor that regulates macrophage activity, can stimulate the excessive production of vascular endothelial growth factor B in macrophages from PVAT and aorta, increase the accumulation of endothelial lipid in obese mice, and promote obesity-induced elevation of blood pressure (95). Eosinophil-deficient ΔdblGATA-1 mice reportedly lack the anti-contractile function of PVAT and show elevated blood pressure (96). Notably, Withers et al. demonstrated that obesity is accompanied by a significant decrease in the number of eosinophils present in PVAT, which may lead to a loss of its anti-contractile function (97). This effect was restored by replenishment using purified eosinophils in vessels with intact PVAT or by the use of IL-33 to restore the number of eosinophils in PVAT (96, 97). These findings suggest that PVAT releases an eosinophil-derived soluble anti-contractile factor. This factor is dependent on B3 adrenoceptor activity and independent of other downstream signaling pathways (e.g., APN and nitric oxide) mediated by immune cells (97). Thus, targeting the number of eosinophils in PVAT may constitute a novel method for the treatment of obesity-related hypertension.

### Diabetes Mellitus-Related Vascular Dysfunction

The progression of DM eventually involves the development of chronic vascular complications and associated cardiovascular diseases; this cardiovascular disease progression is the leading cause of death in diabetic patients worldwide (131). Endothelial dysfunction is the initial vascular defect in DM; it is associated with DM-related macrovascular and microvascular complications (e.g., coronary heart disease, stroke, peripheral vascular disease, diabetic retinopathy, and kidney disease), which represent the main health burden in patients with DM (132). Inflammation is a major pathophysiological process that mediates DM-related endothelial dysfunction. PVAT is presumed to serve as a mechanistic link between T2DM and vascular diseases such as atherosclerosis (133).

In the context of DM, high glucose stimulation induces PVAT transition to a pro-inflammatory (increased CRP, CCL2, and CD36), pro-oxidant, and vasoconstrictive phenotype (98, 99). PVAT inflammation can promote insulin resistance in the vascular system, resulting in impaired insulin-mediated vasodilation and vascular remodeling and subsequent onset of vascular diseases (99). PVAT obtained from obese db/db mice greatly impaired insulin-mediated vasodilation of the resistance artery in muscle, while PVAT obtained from nonobese mice promoted vasodilation of this artery (100). Furthermore, obesity and the expansion of PVAT in db/db mice cause elimination of insulin-stimulated vasodilation and recovery by blocking inflammation through inhibition of the c-Jun N-terminal kinase pathway, thus indicating a key role for inflammation in PVAT (100).

### Abdominal Aortic Aneurysm (AAA)

Inflammatory cell recruitment to aortic media, macrophage activation, and pro-inflammatory molecule production are important mechanisms involved in AAA (134), which contribute to gradual thinning of the aortic media and adventitia (135). Analysis of samples from human patients has shown that AAAs are surrounded by abundant PVAT (103), and the density of PVAT is higher around the aneurysm sac in patients with aortic aneurysm than in healthy neck tissue, suggesting that the deposition of PVAT is related to AAA pathophysiology (104). Overall, PVAT plays a pro-inflammatory role in the development of AAA.

Genome-wide expression profiling has revealed colocalization of PVAT inflammation with AAA, suggesting that biological changes in PVAT may be functionally associated with AAA pathogenesis (105). Changes in PVAT phenotype and function initiate inflammatory signals, stimulating the recruitment and activation of immune cells; the soluble factors produced by immune cells cause matrix degradation, leading to the initiation and progression of AAA (105). PVAT samples from AAA patients showed increased expression of various pro-inflammatory genes, including IL-8, protein tyrosine phosphatase receptor type C, lymphocyte-specific protein tyrosine, and CCL5, accompanied by decreased expression of anti-inflammatory genes (e.g., peroxisome proliferator-activated receptor gamma) and increased degradation of extracellular matrix (106). Furthermore, adipose tissue from AAA patients can induce inflammation in healthy VSMCs from control patients, resulting in increased expression of genes involved in aneurysm formation (106). Studies in animal models have shown that PVAT-derived pro-inflammatory factors accelerate the recruitment of macrophages, lymphocytes, and mast cells in the vascular wall (93). Deletion of the AT1a receptor gene in PVAT attenuated AAA development and gelatinolytic activity, as well as the accumulation of macrophages in abdominal aorta and adipose tissue; it also contributed to macrophage polarization from a pro-inflammatory state to an anti-inflammatory state. In addition, obesity-related PVAT dysfunction reportedly promotes Ang II-induced aortic aneurysm formation by secreting platelet-derived growth factor-D (PDGF-D). Leukocyte–fibroblast interactions in adventitia enhance the recruitment and activation of local monocytes, leading to aortic aneurysm and aortic dissection (107, 108); PDGF-D stimulates the migration and proliferation of adventitial fibroblasts, as well as the expression of pro-inflammatory factors. Notably, adipocyte-specific PDGF-D transgenic mice were more likely to form aortic aneurysm after Ang II infusion, accompanied by increased adventitial inflammation and fibrosis (109). Although multiple studies have shown that macrophages are the key inflammatory cells mediating the formation of AAA (110), immunophenotypic analysis of advanced AAA samples infiltrating the largest expansion site demonstrated that T cells (rather than macrophages) are the main leukocyte subset in AAA; their greatest accumulation occurs in perivascular tissues such as PVAT (111). This discrepancy may be related to differences in AAA stages between studies. However, these findings clearly indicate that inflammation in PVAT and aortic wall contributes to the pathophysiology of AAA; these proposed pathways of inflammatory induction can reveal new therapeutic targets for AAA.

## Conclusion

PVAT dysfunction is one of the main risk factors for cardiovascular diseases; PVAT is particularly important because of its close proximity to the vascular wall. Therefore, the importance of PVAT in regulating cardiovascular complications cannot be ignored. Further mechanistic research is needed; however, immune dysfunction (i.e., increased presence of pro-inflammatory mediators, rather than anti-inflammatory mediators) and subsequent chronic inflammation play key roles. Crosstalk between PVAT and vascular system occurs in both directions, and it has important roles in vascular homeostasis and disease. In particular, inflammation leads to PVAT dysfunction through inflammatory cells and various pro-inflammatory factors, thereby exacerbating altered vasodilation, while enhancing vasoconstriction and vascular remodeling; these changes contribute to vascular aging, atherosclerosis, hypertension, and other vascular diseases. In animal and human studies, PVAT dysfunction has been shown to cause various inflammatory vascular diseases, and vascular inflammation is associated with changes in PVAT phenotype; these findings can help to identify vulnerable vascular lesions. Although the mechanism is not entirely clear, the existing evidence shows that PVAT inflammation is a strictly regulated process that occurs in the early stage of vascular disease, which can serve as a valuable target for future treatment. Therefore, further research is needed to explore whether PVAT can be targeted in novel treatments for vascular diseases.

In addition, PVAT-related secretory factors (e.g., adipokines, hormones, and other factors) have important effects on many aspects of the vascular system. PVAT dysfunction promotes the dedifferentiation of perivascular adipocytes, such that they no longer serve as lipid storage cells; in contrast, they become metabolically active synthetic tissues, produce pro-inflammatory cytokines and chemokines, and play key roles in cardiovascular disease-related inflammation (5). Overall, extensive analysis of various adipokines is needed to clearly distinguish the physiological and therapeutic effects of these adipokines in the context of vascular dysfunction.

## Author Contributions

YL and YZ designed the manuscript and wrote part of it, while YC wrote most of it. ZQ revised and corrected the manuscript, and YW and XL completed the design and drawing of figure and table. All authors contributed to the article and approved the submitted version.

## Conflict of Interest

The authors declare that the research was conducted in the absence of any commercial or financial relationships that could be construed as a potential conflict of interest.

## Publisher’s Note

All claims expressed in this article are solely those of the authors and do not necessarily represent those of their affiliated organizations, or those of the publisher, the editors and the reviewers. Any product that may be evaluated in this article, or claim that may be made by its manufacturer, is not guaranteed or endorsed by the publisher.
